# Outcomes after early and delayed rehabilitation for exacerbation of chronic obstructive pulmonary disease: a nationwide retrospective cohort study in Japan

**DOI:** 10.1186/s12931-017-0552-7

**Published:** 2017-04-21

**Authors:** Hiroki Matsui, Taisuke Jo, Kiyohide Fushimi, Hideo Yasunaga

**Affiliations:** 1 Department of Clinical Epidemiology and Health Economics, School of Public Health, The University of Tokyo, 7-3-1 Hongo, Bunkyo-ku, Tokyo, 113-0033 Japan; 2 Department of Health Services Research, Graduate School of Medicine, The University of Tokyo, 7-3-1 Hongo, Bunkyo-ku, Tokyo, 113-0033 Japan; 3 Department of Health Policy and Informatics, Graduate School of Medicine, Tokyo Medical and Dental University, 1-5-45 Yushima, Bunkyo-ku, Tokyo, 113-8510 Japan

**Keywords:** COPD exacerbation, Pulmonary rehabilitation, Clinical epidemiology

## Abstract

**Background:**

The effectiveness of early pulmonary rehabilitation (PR) for exacerbation of chronic obstructive pulmonary disease (COPD) remains controversial. The present study aimed to compare the outcomes between early and delayed PR for exacerbation of COPD, using a national inpatient database.

**Methods:**

Using the Japanese Diagnosis Procedure Combination database, we examined patients who were transported to hospital for exacerbation of COPD, received PR during hospitalisation, and were discharged to their home. The patients were divided into those who received early PR (defined as PR starting within 48 h of admission) and those who received delayed PR. The outcomes included 90-day readmission, length of stay (LOS), and activities of daily living (Barthel index ≥15) at discharge. Multiple imputation was used for missing data. To assess the associations between early PR and the outcomes, we used risk-adjusted treatment effects and instrumental variable methods.

**Results:**

We identified 12,572 eligible patients, including 8459 patients with delayed PR and 4113 with early PR. In the risk-adjusted treatment effect models, the early PR group had lower proportion of 90-day readmission (risk difference, −3.4%; 95% CI, −5.7% to −1.5%) and shorter LOS (−9.8 days; 95% CI, −10.8 days to −8.7 days) than the delayed PR group. There was no significant difference in activities of daily living at discharge between the two groups. The instrumental variable analyses showed similar results.

**Conclusions:**

In this national database study, early PR was associated with reduced 90-day readmission and shortened LOS in patients with exacerbation of COPD.

## Background

Exacerbation of chronic obstructive pulmonary disease (COPD) can cause frequent unplanned hospitalisations, which potentially result in death and functional disability [1–3].

The effects of early pulmonary rehabilitation (PR) for unstable COPD patients remain controversial. Several previous small-size randomised controlled trials (RCT) showed that early PR reduced readmission and all-cause mortality, and improved patient exercise capability and quality of life [4]. However, recent RCTs produced conflicting results [5, 6].

Although clinical RCTs remain the gold standard for assessing the efficacy of healthcare services, they can only measure the “efficacy” of an intervention under ideal and controlled circumstances. Therefore, the “effectiveness” of early PR for unstable COPD patients remains unclear in routine clinical settings with more heterogeneous populations and less-standardized treatment protocols.

Using a national inpatient database in Japan, the present study aimed to estimate the real-world effectiveness of early PR for exacerbation of COPD compared with delayed PR.

## Methods

### Data source

For the present retrospective cohort study, we used the Diagnosis Procedure Combination database, a Japanese national inpatient database. The database contains administrative claims data and discharge abstracts, and has information on dates and doses of drugs used and daily records of examinations and procedures (including rehabilitation). The database includes the following data: type of admission (planned or unplanned); ambulance service use; and patients’ main diagnoses, comorbidities at admission, and complications after admission. All diagnoses are coded with International Classification of Diseases and Related Health Problems 10th revision (ICD-10) codes. A previous validation study showed good sensitivity and excellent specificity of diagnoses in the database [7]. The database also contains the following detailed patient information: age; sex; body height and weight; smoking index; severity of dyspnoea at admission, based on the Hugh-Jones dyspnoea scale (grades I to V) [8]; level of consciousness at admission, based on the Japan Coma Scale (JCS) [9]; and activities of daily living (ADL) at admission and discharge, based on the Barthel index (0–20) [10].

### Patient selection

We retrospectively collected patients who were admitted to hospitals with a diagnosis of COPD (ICD-10 codes: J41–J44) as the main diagnosis or diagnosis at admission and discharged between 1 July 2010 and 31 December 2013. The readmission records of the identified patients were followed from 1 July 2010 to 31 March 2014. We included patients who met all of the following criteria: (i) primary diagnosis of COPD at admission; (ii) unplanned admission by ambulance service use; (iii) receipt of PR during hospitalisation; (iv) and discharge to their home. For patients with two or more hospitalisations, we only included data for the initial hospitalisation. We excluded patients who were hospitalised for more than 180 days. We compared the basic characteristics of the included and excluded patients.

### Early PR

We defined early PR as any type and intensity of PR started within 48 h of admission. Delayed PR was defined as any type of PR started after 48 h of admission. In Japan, rehabilitation is reimbursed by public health insurance. In general, geriatric PR includes physical therapy that mainly focuses on improving physical function, i.e., PR that provides early ambulation and adaptive or assistive exercises to assist patients in standing, balancing, and walking better [11].

### Outcomes

The primary outcome was readmission within 90 days of discharge. We used length of stay (LOS) as a secondary outcome variable. Because LOS had a skewed distribution, we used the log-transformed LOS. A previous study showed that a Barthel Index score of <15 was a strong predictor of in-hospital mortality in patients with COPD [12]. Therefore, the present study used Barthel Index ≥ 15 at discharge as another secondary outcome.

### Covariates

We compared the following covariates between the early and delayed PR groups: age; sex; Hugh-Jones dyspnoea scale; JCS on admission; Charlson comorbidity index calculated by recorded ICD-10 codes [13]; ADL at admission; smoking index (defined as number of cigarettes smoked per day multiplied by number of years smoked); use of corticosteroid on day of admission (converted to equivalent dose of prednisolone [14]); use of mechanical ventilation on day of admission; use of oxygen on day of admission; intensive care unit admission; and all-cause pre-admission history within 180 days prior to admission. According to a previous study [15], we divided body mass index into the following five categories: <18.5, 18.5–22.9, 23.0–24.9, 25.0–29.9, and ≥30 kg/m^2^. Because a previous study showed that annual case-volume of COPD patients was associated with outcomes [16], we included annual hospital volume of COPD admissions in the analysis. We also included population density of patient neighbourhood area and distance between patient residence and the hospital as proxies for the local organization of health care provision. To understand the details of the rehabilitation programme, we summarized data on total PR sessions (hours) and PR duration (days) for each group.

A standardized difference between the early and delayed PR groups was calculated for all covariates, with values of >10% defined as out of balance [17, 18].

### Statistical analysis

#### Risk-adjusted treatment effect

We estimated the risk-adjusted treatment effect of early PR as the difference between the risk-adjusted outcomes of each treatment arm [19, 20] (known as predictive margins, model-adjusted means, or g-formula). For this estimation, we conducted the following steps: (i) construction of a prediction model for each outcome including all covariates (except total PR sessions and duration of PR) and early PR as the predictors; (ii) setting of the PR status for each arm; (iii) calculation of predicted probabilities or values with the distribution of the covariates in our study population; and (iv) calculation of differences in the predicted probabilities (or values) between the arms. We estimated standard errors with the cluster bootstrap method [21].

#### Instrumental variable analysis

Because the risk-adjusted treatment effects could be biased by unmeasured confounders, we conducted instrumental variable analyses. In general, instrumental variables meet the following criteria: (i) not associated with patient background characteristics; (ii) associated with treatment selection; and (iii) not directly associated with outcomes [22, 23]. For this study, we used the differential distance (DD) [24, 25] as an instrumental variable. DD was defined as the difference between the distance from patient home to nearest hospital (d1) and the distance from patient home to nearest hospital conducting early PR for half of COPD patients (d2); that is, DD equals d1–d2. We divided DD into two categories: 0 and >0. The adjusted treatment effect was estimated by the ordinary least square (OLS) model with all covariates and the two-stage least square (2SLS) model with all covariates and DD. The validity of the instrumental variable was tested by F-statistics and the Hausman specification test. The null hypothesis for the F-statistics was that the instrumental variable was not associated with treatment selection. We investigated whether the instrumental variable met the above criterion (ii). In the present study, the null hypothesis for the Hausman test was that early PR was not endogenous. When the null hypothesis was rejected, early PR was regarded as endogenous, and we adopted the results of the 2SLS model. When the null hypothesis was not rejected, early PR was not regarded as endogenous, and we adopted the results of the OLS model because the variance of the instrumental variable estimator is generally larger than that of the OLS estimator [20].

#### Multiple imputation

We performed multiple imputation for missing data on several variables, because complete-case analyses (excluding all patients with missing data) can lead to biased results. We created 50 multiple imputed datasets by chained equations with the *mice* package of the R Software [26]. All the covariates (except total PR sessions and duration of PR) and outcome variables were included in the data imputation process. We combined all the results and variances based on Rubin’s Rule [27]. The *P*-value in each imputed dataset was combined by z-transformation [28]. We also performed complete-case analyses for comparison.

## Results

**Fig. 1:**
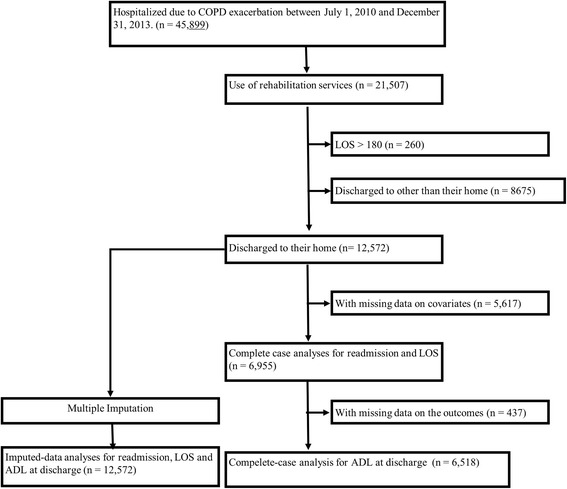
Patient selection

Among all of the patients hospitalized owing to COPD exacerbation during the study period (*n* = 45,899), we excluded those who did not receive rehabilitation, those with LOS >180 days, and those who were discharged to a place other than home. Finally, we identified 12,572 eligible patients during the study period. After exclusion of patients with missing data, there were 6955 patients (Fig. 1).

Appendix 1 Table 5 shows the difference between the included and excluded patients. The excluded patients were more likely to have severe consciousness disorders, a lower BMI, severe physical dependence, a lower Barthel index and a lower Hugh-Jones dyspnoea scale score.

Table 1 presents the characteristics of the eligible patients in the early PR group (*n* = 8459) and delayed PR group (*n* = 4113). The patients in the early PR group were significantly older, had lower Hugh-Jones dyspnoea scale scores, and were more likely to be treated in hospitals with larger annual hospital volumes of COPD patients than those in the delayed PR group. The average number of total PR sessions was slightly higher and the duration of PR was slightly shorter in the early PR group, but the standardized differences were <10%, indicating a well-balanced distribution between the groups.

**Table 1:** Patient baseline characteristics by early pulmonary rehabilitation and differential distance

	Delayed pulmonary rehabilitation	Early pulmonary rehabilitation	Standardized difference (%)
	(*n* = 8459)	(*n* = 4113)	
Age (y), mean (SD)	77.9 (9.5)	79.4 (9.3)	15.9
Sex (female), n (%)	1646 (19.5)	709 (17.2)	5.7
Pre-admission within 180 days, *n* (%)	1951 (23.1)	902 (21.9)	2.7
Japan Coma Scale, *n* (%)			4.5
Alert	6415 (75.8)	3070 (74.6)	
Dull	1453 (17.2)	745 (18.1)	
Somnolence	339 (4.0)	190 (4.6)	
Coma	252 (3.0)	108 (2.6)	
Body mass index, *n* (%)			4.1
< 18.5	2896 (34.2)	1476 (35.9)	
18.5–22.9	2817 (33.3)	1362 (33.1)	
23.0–24.9	701 (8.3)	340 (8.3)	
25.0–29.9	560 (6.6)	257 (6.2)	
≥ 30	115 (1.4)	55 (1.3)	
Missing	1370 (16.2)	623 (15.1)	
Smoking index, mean (SD)	701 (1420)	758 (1857)	3.4
Barthel index, *n* (%)			4.8
0	2935 (34.7)	1400 (34.0)	
5–9	962 (11.4)	460 (11.2)	
10–14	1068 (12.6)	580 (14.1)	
15–19	488 (5.8)	253 (6.2)	
20	1209 (14.3)	566 (13.8)	
Missing	1797 (21.2)	854 (20.8)	
Hugh-Jones dyspnoea scale score, *n* (%)			16.2
0	1212 (14.3)	706 (17.2)	
1	310 (3.7)	203 (4.9)	
2	628 (7.4)	381 (9.3)	
3	787 (9.3)	392 (9.5)	
4	1886 (22.3)	937 (22.8)	
5	3044 (36.0)	1287 (31.3)	
Missing	592 (7.0)	207 (5.0)	
Charlson comorbidity index, *n* (%)			7.5
0–1	5202 (61.5)	2677 (65.1)	
2–3	2839 (33.6)	1247 (30.3)	
≥ 4	418 (4.9)	189 (4.6)	
Intensive care unit admission, *n* (%)	694 (8.2)	398 (9.7)	5.2
Oxygen intake at admission, *n* (%)	5895 (69.7)	2904 (70.6)	2
Intubation at admission, *n* (%)	364 (4.3)	116 (2.8)	8
Steroid dose (mg), mean (SD)	222 (653)	188 (549)	5.6
Hospital volume, mean (SD)	215 (132)	233 (131)	13.9
Population density of patient living area (persons/km^2^), *n* (%)			
< 250	1281 (15.1)	632 (15.4)	1.2
≥ 250	7133 (84.4)	3462 (84.9)	
Missing	45 (0.5)	19 (0.5)	
Distance between patient residence and hospital (km), mean (SD)	6.8 (20.8)	7.7 (34.4)	3.2
Differential distance, *n* (%)			31.5
0 km	3782 (44.7)	1226 (29.8)	
> 0 km	4592 (44.3)	2554 (69.4)	
Missing	85 (1.0)	33 (0.8)	
Total pulmonary rehabilitation session (hours), mean (SD)	7.6 (11.9)	8.5 (12.3)	7.4
Duration of pulmonary rehabilitation (days), mean (SD)	19.2 (21.5)	17.4 (18.5)	9.0

*SD* standard deviation

Appendix 2 Table 6 shows the patient characteristics in the groups with DD of 0 km or >0 km. The patient characteristics were well-balanced with standardized differences of <10.

Table 2 shows the crude outcomes in the delayed and early PR groups.

**Table 2:** Crude outcomes in the delayed and early pulmonary rehabilitation groups

	Delayed pulmonary rehabilitation (*n* = 8459)	Early pulmonary rehabilitation (*n* = 4113)	*P*-value
90-day readmission, *n* (%)	1843 (21.8)	809 (19.7)	0.007
Barthel index ≥15 at discharge, *n* (%)	4193 (49.6)	2027 (49.3)	0.776
Length of stay, mean (SD)	30.4 (24.6)	20.3 (18.5)	<0.001

*SD* standard deviation

Table 3 shows the risk-adjusted treatment effects of early PR on the outcomes. Compared with the delayed PR group, the early PR group had lower 90-day readmission (risk difference, −2.1%; 95% confidence interval (CI), −3.7% to −0.5%) and shorter LOS (difference in LOS, −9.8 days; 95% CI, −10.7 days to −8.8 days) with adjustment for patient characteristics. There was no significant difference in the proportions of Barthel index ≥15 at discharge between the early and delayed PR groups (risk difference, −0.5%; 95% CI, −2.2% to 1.1%).

**Table 3:** Risk-adjusted treatment effects of early pulmonary rehabilitation on the outcomes

	Risk-adjusted treatment effect^a^(95% confidence interval)	*P*-value
90-day readmission (%)	−2.1 (−3.7, −0.5)	0.009
Barthel index ≥15 at discharge (%)	−0.5 (−2.2, 1.1)	0.504
Length of stay (days)	−9.8 (−10.7, −8.8)	<0.001

^a^Adjusted for age, sex, pre-admission within 180 days, Japan coma scale, body mass index, smoking index, Barthel index, Hugh-Jones dyspnoea scale score, Charlson comorbidity index, intensive care unit admission, steroid dose, hospital volume, population density of patient living area, and distance between patient residence and hospital

Table 4 shows the adjusted coefficient estimators of each outcome for early PR in the OLS and 2SLS models. The partial F-statistics from the first-stage regression for each model indicated that DD had sufficient strength for predicting early PR. Early PR was significantly associated with 90-day readmission (coefficient = −0.021; 95% CI, −0.036 to −0.005; *P* = 0.009) in the OLS model, but not in the 2SLS model. The Hausman specification test did not reject the null hypothesis that early PR was exogenous, and we adopted the results of the OLS model. Early PR was significantly associated with log-transformed LOS in both the OLS model (coefficient = −0.424; 95% CI, −0.463 to −0.386; *P* < 0.001) and 2SLS model (coefficient = −0.934; 95% CI, −1.156 to −0.712; *P* < 0.001). The Hausman specification test rejected the null hypothesis that early PR was exogenous, and we adopted the results of the 2SLS model. Early PR was not significantly associated with ADL at discharge in both the OLS model (coefficient = −0.009; 95% CI, −0.025 to 0.007; *P* = 0.264) and 2SLS model (coefficient = 0.059; 95% CI, −0.057 to 0.174; *P* = 0.3188). The Hausman specification test did not reject the null hypothesis that early PR was exogenous, and we adopted the results of the OLS model.

**Table 4:** Ordinary least square and two-stage least square estimates of the outcomes with multiple imputation

	90-day readmission		Barthel index at discharge		Log-transformed length of stay	
	Coefficient (95% CI)	*P*-value	Coefficient (95% CI)	*P*-value	Coefficient (95% CI)	*P*-value
Ordinary least square	−0.021 (−0.036, −0.005)	0.0092	−0.009 (−0.025, 0.007)	0.2644	−0.424 (−0.463, −0.386)	<0.0001
Two-stage least square	−0.053 (−0.159, 0.053)	0.3269	0.059 (−0.057, 0.174)	0.3188	−0.934 (−1.156, −0.712)	<0.0001
F statistics	247.8	<0.0001	247.8	<0.0001	247.8	<0.0001
Hausman specification test		0.559		0.217		<0.0001

*CI* confidence interval

The results of the complete-case analyses are shown in Appendix 3 Tables 7 and 8. These results were similar to those in the imputed analyses.

## Discussion

In this national database study, we showed that early PR was associated with lower proportion of 90-day readmission and shorter LOS in patients with exacerbation of COPD. There was no significant difference in the proportions of Barthel index ≥15 between the early PR and delayed PR groups. Our instrumental variable analyses showed that early PR was significantly associated with reduced 90-day readmission and shortened LOS, but not significantly associated with ADL at discharge.

Because our dataset has several missing values, we conducted multiple imputation analyses. Although our analyses hypothesised the missing at random assumption, our complete-case analyses had similar results to the imputed analyses. This indicates the robustness of our analyses.

Previous small-size RCTs showed that early PR improved patient exercise capacity measured by the 6-min walking test [4, 29] and readmission rate [4, 30]. However, recent large RCTs did not show significant improvement of readmission rate [5, 6],, but did find escalation of the 1-year mortality rate [6]. These disparities between the studies can be explained by the following two aspects: (i) early PR did not have any effect because of short LOS and training duration and (ii) some other factors (e.g. outpatient PR) could have confounded or mediated between the early and delayed PR groups [31]. Our findings showed that early PR was associated with shorter LOS and lower proportion of 90-day readmission after discharge, possibly because the average LOS in Japan is generally longer than those in other developed nations. However, the effect size on 90-day readmission (about 2%) was relatively smaller than that in a previous RCT [6] and expected in the sample size calculation (15%). These findings suggest that the sample sizes in the previous RCTs were too small to have sufficient statistical power for estimating the efficacy of early PR.

As expected, early PR was associated with shorter LOS and lower 90-day readmission rate without worsened ADL status at discharge. Prolonged hospitalisation can reduce patient exercise capacity and may lead to a high probability of readmission [1]. Our results showed that early PR could prevent such adverse effects of prolonged hospitalisation.

Our results indicate that early PR can reduce LOS and readmission rate. Short LOS can improve patient quality of life and reduce hospitalisation costs. A previous study showed that readmission was one of the prognostic factors for COPD [1]. Although the American Thoracic Society recommended early PR for unstable COPD patients, this recommendation was based on an old small-size RCT [2]. Our findings provide new evidence for early PR in unstable COPD patients. Meanwhile, although early PR reduced 90-day readmission, the effect size was smaller than that in a previous RCT and expected in the sample size calculation [6]. Other factors may affect the readmission rate, such as post-discharge outpatient rehabilitation [32].

The population excluded from our study had more severe consciousness disorders, lower BMIs, and more severe physical dependence. The excluded population included those who did not receive rehabilitation and those who were discharged to a place other than home, and many of these people may have been bedridden.

The present study has some strengths. First, we used a large nationwide inpatient database. Patients with exacerbation of COPD who want to participate in randomised rehabilitation trials are not common in daily clinical settings [4]. A previous RCT could not recruit a sufficient population to detect the effects of early PR [6]. Second, we used instrumental variable and missing value imputation analyses to adjust unmeasured confounders for treatment selection and bias from missing values. Third, we used real-world data from a nationwide inpatient database. The present study verified the overall effectiveness of early rehabilitation compared with non-early rehabilitation for patients with acute exacerbation of COPD in a nationwide, real-world clinical setting.

There are also some limitations to the present study. First, our data did not contain post-discharge long-term outcomes. Therefore, we cannot completely detect post-discharge readmission and deaths. Second, we excluded patients who did not receive PR. This may be limit the generalisability of our study. Third, because of data limitations, we could not analyse the details of the PR programmes. The standardized differences in total PR sessions and duration of PR between the early and delayed groups were <10%. Fourth, the Barthel index, one of our secondary outcomes, may not be the most appropriate measure to evaluate the effect of PR. However, the database did not include other short-term outcomes [33].

## Conclusion

In summary, early PR was associated with reduced 90-day readmission and shortened LOS without worsened ADL in patients with exacerbation of COPD. These findings suggest that early PR should be conducted in patients with exacerbation of COPD.

## Appendices

ᅟ

## Appendix 1

**Table 5:** Patient characteristics in the groups included into or excluded from study

	Excluded	Included	Standardized difference (%)
	*n* = 33327	*n* = 12572	
Age (years), mean (sd)	78.3 (10.7)	78.4 (9.5)	1.0
Sex (female), *n* (%)	6415 (19.2)	2355 (18.7)	1.3
Pre-admission within180 day, *n* (%)	7997 (24.0)	2853 (22.7)	3.1
Japan Coma Scale, *n* (%)			20.0
Alert	22972 (68.9)	9485 (75.4)	
Dull	6234 (18.7)	2198 (17.5)	
Somnolence	2006 (6.0)	529 (4.2)	
Coma	2114 (6.3)	360 (2.9)	
Missing	1 (0.0)	0 (0.0)	
Body mass index, *n* (%)			13.1
< 18.5	11733 (35.2)	4372 (34.8)	
18.5–22.9	10081 (30.2)	4179 (33.2)	
23.0–24.9	2440 (7.3)	1041 (8.3)	
25.0–29.9	1926 (5.8)	817 (6.5)	
≥ 30	349 (1.0)	170 (1.4)	
Missing	6798 (20.4)	1993 (15.9)	
Smoking index, mean (sd)	4218 (596790)	719 (1577)	0.8
Barthel index, *n* (%)			15.5
0	13601 (40.8)	4335 (34.5)	
5–9	2928 (8.8)	1422 (11.3)	
10–14	3565 (10.7)	1648 (13.1)	
15–19	1882 (5.6)	741 (5.9)	
20	4910 (14.7)	1775 (14.1)	
Missing	6441 (19.3)	2651 (21.1)	
Hugh-Jones dyspnoea scale score, n (%)			19.6
0	7076 (21.2)	1918 (15.3)	
1	1494 (4.5)	513 (4.1)	
2	2412 (7.2)	1009 (8.0)	
3	2775 (8.3)	1179 (9.4)	
4	5624 (16.9)	2823 (22.5)	
5	11574 (34.7)	4331 (34.4)	
Missing	2372 (7.1)	799 (6.4)	
Charlson comorbidity index, *n* (%)			6.8
0–1	20712 (62.1)	7879 (62.7)	
2–3	10494 (31.5)	4086 (32.5)	
≥ 4	2121 (6.4)	607 (4.8)	
Intensive care unit admission, *n* (%)	2093 (6.3)	1088 (8.7)	9.2
Oxygen intake at admission, *n* (%)	22151 (66.5)	8787 (69.9)	7.4
Intubation at admission, *n* (%)	1547 (4.6)	478 (3.8)	4.2
Steroid dose (mg), mean (sd)	180.6 (611.3)	210.6 (620.9)	4.9
Hospital volume, mean (sd)	209.4 (139.4)	221.1 (131.9)	8.6
Population density of patient living area ≤ 249 persons/km^2^, *n* (%)	27697 (83.1)	10595 (84.3)	3.2
Missing	169 (0.5)	64 (0.5)	
Distance between patient residence and hospital (km), mean (SD)	6.0 (19.6)	7.1 (26.0)	4.6

## Appendix 2

**Table 6:** Patient characteristics in the groups with differential distance =0 km or > 0 km

	Differential distance = 0	Differential distance > 0	Standardized difference (%)
	*n* = 7446	*n* = 5008	
Age (years), mean (sd)	78.7 (9.4)	78.1 (9.6)	6.6
Sex (female), *n* (%)	1357 (18.2)	975 (19.5)	3.2
Pre-admission within 180 day, *n* (%)	1695 (22.8)	1120 (22.4)	1
Japan Coma Scale, *n* (%)			4.1
Alert	5604 (75.3)	3784 (75.6)	
Dull	1299 (17.4)	882 (17.6)	
Somnolence	337 (4.5)	189 (3.8)	
Coma	206 (2.8)	153 (3.1)	
Body mass index, *n* (%)			7.6
< 18.5	2644 (35.5)	1684 (33.6)	
18.5–22.9	2431 (32.6)	1710 (34.1)	
23.0–24.9	648 (8.7)	382 (7.6)	
25.0–29.9	493 (6.6)	321 (6.4)	
≥ 30	87 (1.2)	83 (1.7)	
Missing	1143 (15.4)	828 (16.5)	
Smoking index, mean (sd)	713 (1472)	728 (1735)	0.9
Barthel index, *n* (%)			5.1
0	2556 (34.3)	1743 (34.8)	
5–9	858 (11.5)	549 (11.0)	
10–14	1001 (13.4)	626 (12.5)	
15–19	440 (5.9)	298 (6.0)	
20	1010 (13.6)	751 (15.0)	
Missing	1581 (21.2)	1041 (20.8)	
Hugh-Jones dyspnoea scale score, *n* (%)			9.4
0	1182 (15.9)	720 (14.4)	
1	333 (4.5)	178 (3.6)	
2	625 (8.4)	374 (7.5)	
3	718 (9.6)	449 (9.0)	
4	1665 (22.4)	1126 (22.5)	
5	2471 (33.2)	1820 (36.3)	
Missing	452 (6.1)	341 (6.8)	
Charlson comorbidity index, *n* (%)			0.3
0–1	4663 (62.6)	3140 (62.7)	
2–3	2424 (32.6)	1624 (32.4)	
≥ 4	359 (4.8)	244 (4.9)	
Intensive care unit admission, *n* (%)	668 (9.0)	414 (8.3)	2.5
Oxygen intake at admission, *n* (%)	5238 (70.3)	3470 (69.3)	2.3
Intubation at admission, *n* (%)	252 (3.4)	228 (4.6)	6
Steroid dose (mg), mean (sd)	206 (609)	219 (643)	2.2
Hospital volume, mean (sd)	222 (128)	219 (138)	2.4
Population density of patient living area ≤ 249 persons/km^2^, *n* (%)	1147 (15.4)	752 (15.0)	1.1
Distance between patient residence and hospital (km), mean (SD)	6.08 (14.84)	8.58 (36.81)	8.9
Early pulmonary rehabilitation	0.38 (0.49)	0.24 (0.43)	30.2
Rehabilitation total session (hours), mean (sd)	8.3 (12.7)	7.4 (11.0)	7.2
Rehabilitation duration (days), mean (sd)	18.2 (20.0)	19.2 (21.5)	4.8

## Appendix 3

**Table 7:** Risk-adjusted treatment effects of early pulmonary rehabilitation on the outcomes with complete case data

	Risk-adjusted treatment effect^a^ (95% Confidence interval)	*P* value
90-day readmission (%)	−3.4 (−5.7 to −1.5)	0.001
Barthel index ≥15 at discharge (%)	0.18 (−1.7 to 2.2)	0.552
Length of stay (days)	−9.8 (−10.8 to −8.7)	<0.001

^a^Adjusted for age, sex, pre-admission within 180 days, Japan coma scale, body mass index, smoking index, Barthel index, Hugh-Jones score, Charlson comorbidity index, intensive care unit admission, steroid dose, hospital volume, population density of patient living area, and distance between patient residence and hospital

**Table 8:** Two-stage least squares estimates for outcomes with complete case data, adjusted for measured and unmeasured characteristics

	90-day readmission		Barthel index at discharge		Log-transformed length of stay	
	Coefficient (95% CI)	*P* value	Coefficient (95% CI)	*P* value	Coefficient (95% CI)	*P* value
Ordinary least square;	−0.0348 (−0.0561, −0.0135)	0.0014	−0.0009 (−0.0204, 0.0187)	0.9298	−0.4134 (−0.4551, −0.3718)	< 0.0001
Two-stage least square	−0.039 (−0.1867, 0.1088)	0.6052	0.0664 (−0.0796, 0.2125)	0.3727	−0.9087 (−1.1725, −0.6450)	< 0.0001
F statistics	134.8	< 0.0001	117.2	< 0.0001	134.8	< 0.0001
Hausman specification test		0.956		0.339		< 0.0001

*CI* Confidence interval

## Abbreviations

2SLS: Two-stage least square
ADL: Activities of daily living
CI: Confidence interval
COPD: Chronic obstructive pulmonary disease
DD: Differential distance
ICD-10: International Classification of Diseases and Related Health Problems 10th revision (ICD-10)
JCS: Japan Coma Scale
LOS: Length of stay
OLS: Ordinary least square
PR: Pulmonary rehabilitation
RCT: Randomised controlled trials
SD: Standard deviation
